# Fra2 Is a Co-Regulator of Fep1 Inhibition in Response to Iron Starvation

**DOI:** 10.1371/journal.pone.0098959

**Published:** 2014-06-04

**Authors:** Jean-François Jacques, Alexandre Mercier, Ariane Brault, Thierry Mourer, Simon Labbé

**Affiliations:** Département de Biochimie, Faculté de médecine et des sciences de la santé, Université de Sherbrooke, Sherbrooke, Quebec, Canada; University of Cambridge, United Kingdom

## Abstract

Iron is required for several metabolic functions involved in cellular growth. Although several players involved in iron transport have been identified, the mechanisms by which iron-responsive transcription factors are controlled are still poorly understood. In *Schizosaccharomyces pombe*, the Fep1 transcription factor represses genes involved in iron acquisition in response to high levels of iron. In contrast, when iron levels are low, Fep1 becomes inactive and loses its ability to associate with chromatin. Although the molecular basis by which Fep1 is inactivated under iron starvation remains unknown, this process requires the monothiol glutaredoxin Grx4. Here, we demonstrate that Fra2 plays a role in the negative regulation of Fep1 activity. Disruption of *fra2^+^* (*fra2Δ*) led to a constitutive repression of the *fio1^+^* gene transcription. Fep1 was consistently active and constitutively bound to its target gene promoters in cells lacking *fra2^+^*. A constitutive activation of Fep1 was also observed in a *php4Δ fra2Δ* double mutant strain in which the behavior of Fep1 is freed of its transcriptional regulation by Php4. Microscopic analyses of cells expressing a functional Fra2-Myc_13_ protein revealed that Fra2 localized throughout the cells with a significant proportion of Fra2 being observed within the nuclei. Further analysis by coimmunoprecipitation showed that Fra2, Fep1 and Grx4 are associated in a heteroprotein complex. Bimolecular fluorescence complementation experiments brought further evidence that an interaction between Fep1 and Fra2 occurs in the nucleus. Taken together, results reported here revealed that Fra2 plays a role in the Grx4-mediated pathway that inactivates Fep1 in response to iron deficiency.

## Introduction

Iron is required at the active center of several important enzymes, including those involved in the tricarboxylic acid cycle, respiration, lipid metabolism, DNA replication and repair [1], [2]. Because of its ability to lose or gain a single electron, iron is an important cofactor for electron transfer between different donors and acceptors. Paradoxically, iron can be highly toxic when allowed to accumulate in excess. Indeed, high concentrations of iron have the potential to produce toxic hydroxyl radicals through the Fenton reaction [3]. These two facets of iron properties require that organisms must sense their internal iron load and respond appropriately by regulating iron acquisition, thereby keeping iron concentrations under tight control.

Studies using the yeast model *Schizosaccharomyces pombe* have allowed discovery of genes encoding proteins that function in the regulation of iron homeostasis [4]. The GATA-type transcription factor Fep1 represses several genes involved in iron acquisition when iron levels are high [5], [6]. A second iron-responsive factor, denoted Php4, is critical for down-regulating genes encoding iron-using proteins when iron levels are low [7], [8]. Php4 is a subunit of the CCAAT-binding protein complex. In response to iron starvation, Php4 is synthesized and interacts with the Php2/Php3/Php5 heterotrimer to repress genes that encode components of iron-requiring metabolic pathways, such as the tricarboxylic acid cycle, the electron transport chain, and the iron-sulfur cluster biogenesis machinery [7], [8]. When cells undergo transition from iron-limiting to iron-sufficient conditions, *php4^+^* expression is repressed by the iron-dependent transcriptional repressor Fep1. In contrast, when iron levels are low, Php4 is responsible for the transcriptional repression of *fep1^+^*. Thus, Php4 and Fep1 reciprocally control each other's expression as a function of changes in iron levels [4]. Chromatin immunoprecipitation (ChIP) experiments have revealed that Fep1 binds to GATA elements *in vivo* in an iron-dependent manner [9]. In contrast, under iron deficient conditions, Fep1 fails to bind chromatin and that results in markedly increased transcription of genes encoding iron acquisition proteins. Fep1-like transcription factors are widely distributed in other fungi such as *Ustilago maydis*, *Aspergillus nidulans*, *Histoplasma capsulatum*, and *Cryptococcus neoformans*, but not in *Saccharomyces* species [10]–[12].

CGFS-type monothiol glutaredoxins are classified into two groups. The first group is composed of single-domain CGFS monothiol glutaredoxins involved in iron-sulfur protein biogenesis and maturation [13], [14]. The second group consists of multidomain CGFS monothiol glutaredoxins. These glutaredoxins deliver and transfer iron-sulfur clusters to proteins and subcellular compartments [15]. In addition, they sense and communicate cellular iron status to iron-responsive transcription factors [16]–[20]. In *S. pombe*, the multidomain CGFS monothiol glutaredoxin Grx4 harbors two distinct regions, a N-terminal thioredoxin (TRX)-like domain and a C-terminal glutaredoxin (GRX)-like domain. The TRX domain of Grx4 contains a WAAPC^35^K sequence that is similar to the thioredoxin active site motif WCGPCK [21], [22]. Recent studies have suggested that the TRX domain serves as a docking site for interacting partners of multidomain CGFS monothiol glutaredoxins [18], [19], [23]. The GRX domain of Grx4 contains a typical ^172^CGFS^175^ active site motif. The CGFS-type monothiol glutaredoxins can form [2Fe-2S]-bridged homodimers [24]–[26]. The combination of two GRX domains (containing one CGFS motif each) generates two Cys ligands to which a [2Fe-2S] cluster can be coordinated with the aid of two glutathione molecules that provide the other two cluster ligands. This complex results in a glutathione-ligated [2Fe-2S] center that is held within the monothiol glutaredoxin dimer. Inactivation of the *grx4^+^* gene (*grx4Δ*) makes a constitutively active Fep1 that binds to its target gene promoters *in vivo*. In the absence of Grx4, Fep1 behaves like an insensitive protein, constitutively repressing target gene expression [18], [27]. Although the molecular basis by which Grx4 communicates iron deficiency to Fep1 remains obscure, two-hybrid and coimmunoprecipitation experiments have revealed that the TRX domain of Grx4 associates strongly and constitutively with the C-terminal region of Fep1. Subsequent analyses have shown that, under low but not high iron conditions, the GRX domain of Grx4 associates with the N-terminal region of Fep1, which contains its DNA-binding domain. A speculative mechanism for iron starvation-dependent inactivation of Fep1 by Grx4 would be that Fep1-GRX domain interaction triggers conformational changes that impair Fep1 DNA binding, thus blocking its association with chromatin and its repressive effect on target gene expression. Given the fact that Fep1 and multidomain CGFS monothiol glutaredoxins can form homodimers, a dimer of Fep1 may associate with two Grx4 molecules. Under conditions of iron excess, two GRX domains of Grx4 could themselves coordinate a [2Fe-2S] cluster, making the N-terminal region of Fep1 available for interaction with chromatin.

Interestingly, a number of studies have shown that Grx4 plays a role in conveying the information of the presence of iron to Php4 [19], [28]. In fact, disruption of *grx4^+^* (*grx4Δ*) leads to constitutive activation of Php4, making iron-regulated genes that are under its control to be continually repressed, irrespective of cellular iron status. Under high iron conditions, the GRX domain of Grx4 interacts with Php4 in an iron-dependent manner. This association between GRX domain and Php4 fosters the inactivation and release of Php4 from the Php2/Php3/Php5 complex and its subsequent export from the nucleus to the cytoplasm by exportin Crm1 [28]. In contrast, under iron deficiency, the GRX domain dissociates from Php4, allowing Php4 to bind to the Php2/Php3/Php5 heterotrimeric complex, which represses transcription of iron-using genes.

In *S. cerevisiae*, Aft1 is a major iron-responsive transcription factor that activates the expression of genes involved in iron metabolism, including the high-affinity iron uptake genes [29]–[31]. Aft1 *trans*-activates gene expression under iron starvation conditions but its activity is inhibited under iron-replete conditions. A number of studies have shown that the multidomain CGFS monothiol glutaredoxins Grx3 and Grx4 are required for iron-dependent inhibition of Aft1 [16], [32], [33]. Grx3/Grx4-mediated inhibition of Aft1 also involves Fra2 and possibly Fra1, since these two proteins are found as a cytosolic complex with monothiol glutaredoxins (Grx3/Grx4) and Aft1 under iron-replete conditions [34], [35]. The *fra1^+^* gene encodes an aminopeptidase P-like protein, whereas the *fra2^+^* gene encodes a BolA2-like protein, which has been shown to form a [2Fe-2S]-bridged complex with both Grx3 and Grx4 [36]. A current model posits that association between Fra2 and Grx3/Grx4 transmits an as-yet-unidentified mitochondrial inhibitory signal to Aft1 that is dependent on the biosynthesis of mitochondrial iron-sulfur clusters [34], [37], [38]. Upon sensing this signal, an iron-sulfur cluster dependent Grx3/Grx4-Aft1 interaction occurs and favors removal of Aft1 from its target gene promoters, leading to Aft1 inactivation [20].

*S. pombe* contains one Fra1-like and three BolA-like proteins, denoted Uvi31, Fra2 and Fra3, which are predicted to belong to BolA1, BolA2 and BolA3 subfamilies, respectively [32]. Several reports have highlighted essential roles for Fra proteins in the regulation of cellular iron homeostasis [32], [34], [35]. Here, we have tested the possibility that *S. pombe* Fra1-3 and Uvi31 affected Fep1 activity as a function of iron availability. Deletion of *fra2^+^* (*fra2Δ*) caused a constitutive repression of iron transport genes and led to constitutive promoter occupancy by Fep1. In contrast, deletions of *fra1^+^*, *fra3^+^*, and *uvi31^+^* did not cause defects in the transcriptional response to iron starvation. Cells carrying disrupted *fra2Δ php4Δ* alleles engineered to unlink iron-dependent behavior of Fep1 from its transcriptional regulation by Php4, were phenocopies of *fra2Δ* single disruption strain. When coexpressed in fission yeast, Fra2, Grx4 and Fep1 were detected as a heteroprotein complex under both iron-deficient and iron-replete conditions. Further analysis by bimolecular fluorescence complementation (BiFC) assays revealed that Fra2 is a binding partner of Fep1 and their association occur in the nuclear compartment of *S. pombe*. Taken together, our findings indicate that the Grx4-sensing pathway that inactivates Fep1 in response to iron deficiency requires a functional Fra2 protein.

## Materials and Methods

### Strains and growth conditions

*S. pombe* strains used in this study are listed in Table 1. Under nonselective conditions, *S. pombe* cells were grown on yeast extract plus supplement medium (YES) containing 0.5% yeast extract, 3% glucose and 225 mg/l of adenine, histidine, leucine, lysine and uracil. When plasmid integration was required, cells were cultured in Edinburgh minimal medium lacking specific nutrients to isolate cells expressing the integrative plasmid [39]. Liquid cultures were seeded at an *A_600_* of 0.5 and then grown to late exponential phase (*A_600_* of ∼1.0) in the presence of FeCl_3_ (100 µM). After washing, aliquots of cultures were either incubated in the presence of 2, 2′-dipyridyl (Dip) (250 µM) or FeCl_3_ (100 µM or 250 µM), or were left untreated for 30 min, unless otherwise indicated.

**Table 1:** *S. pombe* strain genotypes.

Strain	Genotype	Source or reference
FY435	h^+^ his7-366 leu1-32 ura4-Δ18 ade6-M210	[5]
AMY35	h^+^ his7-366 leu1-32 ura4-Δ18 ade6-M210 fra1Δ::KAN^r^	This study
AMY36	h^+^ his7-366 leu1-32 ura4-Δ18 ade6-M210 fra2Δ::KAN^r^	This study
AMY39	h^+^ his7-366 leu1-32 ura4-Δ18 ade6-M210 fra1Δ::loxP fra2Δ::KAN^r^	This study
AMY43	h^+^ his7-366 leu1-32 ura4-Δ18 ade6-M210 fra3Δ::KAN^r^	This study
AMY44	h^+^ his7-366 leu1-32 ura4-Δ18 ade6-M210 fra1Δ::loxP fra3Δ::KAN^r^	This study
AMY45	h^+^ his7-366 leu1-32 ura4-Δ18 ade6-M210 fra2Δ::loxP fra3Δ::KAN^r^	This study
AMY46	h^+^ his7-366 leu1-32 ura4-Δ18 ade6-M210 fra1Δ::loxP fra2Δ::loxP fra3Δ::KAN^r^	This study
JFJ148	h^+^ his7-366 leu1-32 ura4-Δ18 ade6-M210 uvi31Δ::KAN^r^	This study
JFJ156	h^+^ his7-366 leu1-32 ura4-Δ18 ade6-M210 fra2Δ::loxP uvi31Δ::KAN^r^	This study
JFJ164	h^+^ his7-366 leu1-32 ura4-Δ18 ade6-M210 fra3Δ::loxP uvi31Δ::KAN^r^	This study
JFJ172	h^+^ his7-366 leu1-32 ura4-Δ18 ade6-M210 fra2Δ::loxP fra3Δ::loxP uvi31Δ::KAN^r^	This study
AMY15	h^+^ his7-366 leu1-32 ura4-Δ18 ade6-M210 php4Δ::KAN^r^	[7]
JFJ195	h^+^ his7-366 leu1-32 ura4-Δ18 ade6-M210 fra2^+^-myc13::KAN^r^	This study
JFJ89	h^+^ his7-366 leu1-32 ura4-Δ18 ade6-M210 fra2Δ::loxP php4Δ::KAN^r^	This study
JFJ101	h^+^ his7-366 leu1-32 ura4-Δ18 ade6-M210 fra2Δ::loxP php4Δ::loxP fep1Δ::KAN^r^	This study
fep1Δ php4Δ	h^+^ his7-366 leu1-32 ura4-Δ18 ade6-M210 fep1Δ::KAN^r^ php4Δ::loxP	[9]
fep1Δ php4Δ TAP-fep1^+^	h^+^ his7-366 leu1-32 ura4-Δ18 ade6-M210 fep1Δ::KAN^r^ php4Δ::loxP TAP-fep1^+^::leu1^+^	[9]
fep1Δ php4Δ fep1^+^	h^+^ his7-366 leu1-32 ura4-Δ18 ade6-M210 fep1Δ::KAN^r^ php4Δ::loxP fep1^+^::leu1^+^	[9]
JFJ142	h^+^ his7-366 leu1-32 ura4-Δ18 ade6-M210 fep1Δ::KAN^r^ php4Δ::loxP fra2Δ::loxP TAP-fep1^+^::leu1^+^	This study
fep1Δ php4Δ grx4Δ TAP-fep1^+^	h^+^ his7-366 leu1-32 ura4-Δ18 ade6-M210 fep1Δ::loxP php4Δ::loxP grx4Δ::KAN^r^ TAP-fep1^+^::leu1^+^	[18]
JFJ196	h^+^ his7-366 leu1-32 ura4-Δ18 ade6-M210 fra2Δ::loxP fra2^+^-myc13::KAN^r^	This study
JFJ215	h^+^ his7-366 leu1-32 ura4-Δ18 ade6-M210 grx4Δ::KAN^r^ GFP-Grx4^+^::ade6^+^	This study
fep1Δ fep1^+^-GFP	h^+^ his7-366 leu1-32 ura4-Δ18 ade6-M210 fep1Δ::ura4^+^ fep1^+^-GFP::leu^+^	[45]
JFJ207	h^+^ his7-366 leu1-32 ura4-Δ18 ade6-M210 grx4Δ::KAN^r^ fep1Δ::ura4^+^ GFP-Grx4^+^::ade6^+^ TAP-fep1^+^::leu1^+^	This study
JFJ217	h^+^ his7-366 leu1-32 ura4-Δ18 ade6-M210 grx4Δ::loxP fep1Δ::ura4^+^ fra2Δ::KAN^r^ GFP-Grx4^+^::ade6^+^ TAP-fep1^+^::leu1^+^	This study
JFJ258	h^+^ his7-366 leu1-32 ura4-Δ18 ade6-M210 grx4Δ::loxP fep1Δ::ura4^+^ fra2^+^-myc13::KAN^r^ GFP-Grx4^+^::ade6^+^ TAP-fep1^+^::leu1^+^	This study
JFJ285	h^+^ his7-366 leu1-32 ura4-Δ18 ade6-M210 grx4Δ::loxP fra2^+^-myc13::KAN^r^ GFP-Grx4^+^::ade6^+^ TAP::leu1^+^	This study
JFJ243	h^+^ his7-366 leu1-32 ura4-Δ18 ade6-M210 fep1Δ::ura4^+^ fra2^+^-VC::KAN^r^ VN-fep1^+^::leu1^+^	This study
JFJ241	h^+^ his7-366 leu1-32 ura4-Δ18 ade6-M210 fep1Δ::ura4^+^ VC::ade6^+^ VN-fep1^+^::leu1^+^	This study
JFJ291	h^+^ his7-366 leu1-32 ura4-Δ18 ade6-M210 fep1Δ::ura4^+^ ctr4Δ::loxP ctr4^+^-VC::ade6^+^ VN-fep1^+^::leu1^+^	This study
JFJ309	h^+^ his7-366 leu1-32 ura4-Δ18 ade6-M210 grx4Δ::loxP fra2Δ::KAN^r^	This study

### RNA isolation and analysis

Total RNA was extracted using a hot phenol method as described previously [40]. In the case of the RNase protection assays, RNA (15 µg per reaction) was hybridized with the indicated riboprobes (Table 2), as described previously [8]. DNA templates for antisense riboprobes were cloned into BamHI and EcoRI sites of the pBluescript SK vector (Stratagene, La Jolla, CA). The resulting constructs were linearized with BamHI for subsequent antisense RNA labeling with [α-^32^P]UTP and T7 RNA polymerase. *act1^+^* mRNA was probed as an internal control for normalization during quantification of RNase protection products.

**Table 2:** Riboprobes used to detect steady-state levels of transcripts.

Gene ID	Gene	Riboprobe length (bp)	Position relative to initiator codon	Source or reference
SPBC32H8.12c	*act1^+^*	151	+334 to +485	[7]
SPAC23E2.01	*fep1^+^*	181	+68 to +249	[9]
SPAC1F7.08	*fio1^+^*	218	+91 to +309	[5]
SPAC22G7.01c	*fra1^+^*	175	+1384 to +1558	This study
SPAC8C9.11	*fra2^+^*	179	+3 to +181	This study
SPCC4B3.11c	*fra3^+^*	178	+6 to +183	This study
SPCC645.03c	*isa1^+^*	188	+3 to +191	[7]
SPBC16E9.06c	*uvi31^+^*	178	+53 to +230	This study

### ChIP experiments

Early logarithmic-phase cells (100 ml of each culture) were incubated in the presence of FeCl_3_ (100 µM). At mid-logarithmic phase, the cells were washed twice, divided in 50 ml cultures and then grown in the presence of FeCl_3_ (250 µM) or Dip (250 µM). Formaldehyde was added to a final concentration of 1%, as described previously [9], [41]. After formaldehyde-mediated cross-links and neutralization with glycine, cell lysates were prepared as described previously [41]. Samples were subsequently sonicated 10 times (10 s cycles at 20 amplitude microns [20%]) using a Branson 450 sonicator in order to shear chromatin DNA into fragments of ∼400 to 1000 bp. Immunoprecipitation of TAP-tagged Fep1 bound to chromatin, immunoglobulin G (IgG)-Sepharose beads washings and elution, reversed cross-linking and DNA precipitation were performed as described previously [9], [42]. Quantification of immunoprecipitated DNA was carried out by quantitative real-time polymerase chain reaction (qPCR) using primers that spanned a *fio1* ^+^ promoter region that included functional GATA boxes [5]. TAP-tagged Fep1 density at the a *fio1* ^+^ promoter was calculated as the enrichment of the specific genomic *fio1* ^+^ promoter region relative to a 18S ribosomal DNA coding region in which no GATA box is present. Primers were designated by the name of the gene promoter followed by the position of their 5′ ends relative to that of the translational initiation codon: *fio1-830* (5′-CCCACTTCTTCCAGGCATCTG-3′) and *fio1-741* (5′-GTCGGAGTTGGTGTCCACTTTG-3′). Two primers derived from a 18S ribosomal DNA coding region were used as internal background controls: 18S-a (5′-CAGCTTGCGTTGAATACGTCCC-3′) and 18S-b (5′-AGCCAATCCAGAGGCCTCACTA-3′) [41]. Each qPCR experiment was performed in triplicate, and all ChIP experiments were repeated at least three times using independent chromatin preparations.

### Protein tagging

To create a strain in which the *Myc_13_* or Venus N-terminal fragment (*VN*) coding sequence was integrated at the chromosomal locus of *fra2^+^* (downstream of and in-frame to the 3′ terminal region of *fra2^+^*), a PCR-based gene fusion approach was used as described previously [43], [44]. PCR primers were 100 nucleotides in length and they corresponded to the last 80 nucleotides of *fra2^+^* (without stop codon) (upper strand) and the first 80 nucleotides of the 3′ untranslated region of *fra2^+^* (lower strand). At their 3′ ends, each pair of primers contained sequences corresponding to the first and last 20 nucleotides of pFA6a-13Myc-kanMX6 [43] or pFA6a-Venus C-terminal fragment (VC)-kanMX6 module [44]. The method allowed homologous integration of *Myc_13_* or *VN* at the chromosomal locus of *fra2^+^*, thereby replacing wild-type allele by a *Myc_13_*- or *VN*-tagged *fra2^+^* allele. The *TAP-fep1^+^* allele was constructed as described previously [45] and its integration was performed by homologous recombination at the *leu1^+^* locus.

### Coimmunoprecipitation experiments

To determine whether Fra2 interacted with Fep1 in *S. pombe*, *fep1Δ grx4Δ fra2^+^-Myc_13_* cells were co-transformed with pJB1-194^*^ *promphp4^+^-*green fluorescent protein(*GFP*)*-grx4^+^* and pJK-1478NTAP-*fep1^+^*. Cultures were grown in Edinburgh minimal medium to an *A*_600_ of 1.0 in the presence of FeCl_3_ (100 µM). After washings, aliquots of cultures were either incubated in the presence of Dip (250 µM) or FeCl_3_ (100 µM) for 30 min. Total cell lysates were obtained by glass bead disruption in lysis buffer (10 mM Tris-HCl (pH 7.9), 0.1% Triton X-100, 0.5 mM EDTA, 20% glycerol, 100 mM NaCl and 1 mM phenylmethylsulfonyl fluoride) containing a mixture of protease inhibitors (P-8340; Sigma-Aldrich). After centrifugation at 13,000 rpm at 4°C for 5 min, equal amounts of proteins (∼2.7 mg) were added to 15-µl bed volumes of IgG-Sepharose 6 Fast-Flow beads (GE Healthcare) and the suspensions were end-over-end mixed for 18 h at 4°C. The beads were washed four times with 1 ml of lysis buffer and then transferred to fresh microtubes prior to a final wash. The immunoprecipitates were resuspended in 60 µl of sodium dodecyl sulfate loading buffer, heated for 5 min at 95°C and proteins resolved by electrophoresis on 9-% sodium dodecyl sulfate-polyacrylamide gels. For Western blotting analysis of Fra2-Myc_13_, TAP-Fep1, GFP-Grx4 and α-tubulin, the following antibodies were used: monoclonal anti-*c*-*myc* antibody 9E10 (Roche Diagnostics); polyclonal anti-mouse IgG antibody (ICN Biomedicals); monoclonal anti-GFP antibody B-2 (Santa Cruz Biotechnology); and, monoclonal anti-α-tubulin antibody (clone B-5-1-2; Sigma-Aldrich). Following incubation with primary antibodies, membranes were washed and incubated with the appropriate horseradish peroxidase-conjugated secondary antibodies (Amersham Biosciences), developed with enhanced chemiluminescence reagents (Amersham Biosciences), and visualized by chemiluminescence.

### Direct and indirect immunofluorescence microscopy

Microscopic analyses of GFP-Grx4 and Fep1-GFP fusion proteins were carried out as described previously [45], except that cells were incubated in the presence of FeCl_3_ or Dip for 30 min. For localization of a functional Myc_13_ epitope-tagged Fra2 protein, indirect immunofluorescence microscopy was performed as described previously [46], except that cells were fixed with formaldehyde (methanol-free) after a 30-min incubation in the absence or presence of FeCl_3_ (100 µM) or Dip (250 µM).

### BiFC analysis

Analysis of VN-Fep1, Fra2-VC and Ctr4-VC fusion proteins was performed using BiFC assays as described previously [47]. Fluorescence and differential interference contrast images of the cells were obtained using an Eclipse E800 epifluorescent microscope (Nikon, Melville, NY) equipped with an ORCA ER digital cooled camera (Hamamatsu, Bridgewater, NJ). BiFC signals were visualized using a magnification of x1000 with a transmission window of 465 to 495 nm, whereas chromosomal material (Hoechst 33342-staining) was detected with a window of 340 to 380 nm. The cell fields shown in this study represent a minimum of five independent experiments. The merged images were obtained using the Simple PCI software, version 5.3.0.1102 (Compix, Sewickly, PA).

## Results

### Effect of disrupting *fra1^+^* and bolA-like genes on the Fep1 target gene *fio1^+^*

**Figure 1:**
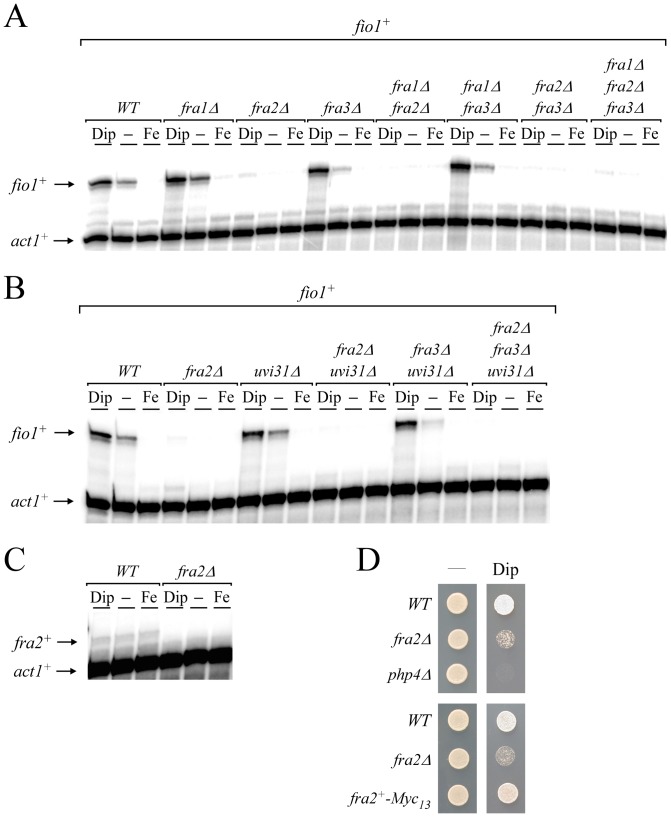
Disruption of *fra2^+^* renders cells unable to induce *fio1^+^* gene expression in response to iron starvation. *A*, Wild-type (*WT*) and the indicated isogenic mutant strains were left untreated (-) or were treated with Dip (250 µM) or FeCl_3_ (Fe, 100 µM) for 30 min. Total RNA was extracted and *fio1^+^* and *act1^+^* steady-state mRNA levels were analyzed by RNase protection assays. *B*, The indicated isogenic strains were cultured as described in *panel A*. Total RNA was isolated and analyzed for steady-state levels of *fio1^+^* and *act1^+^* mRNAs. *C*, *fra2^+^* steady-state mRNA levels were analyzed in the absence (-) or presence of Dip (250 µM) or FeCl_3_ (Fe, 100 µM). No *fra2^+^* transcript was observed in the *fra2Δ* mutant strain. Data shown in *panels A*, *B* and *C* are representative of three independent experiments. *D*, The isogenic wild-type (*WT*), *fra2Δ*, and *php4Δ* strains were spotted onto YES medium containing none (-) or 150 µM Dip and incubated at 30°C for 5 days (*top panel*). The *php4Δ* mutant was used as a control strain known to be hypersensitive to Dip. Cells expressing a functional *fra2^+^-Myc_13_* allele were spotted onto YES medium that was either supplemented or not with Dip (150 µM) (*bottom panel*).

Our previous studies showed that Grx4 was required to inhibit the iron-dependent repressor Fep1 when cells underwent transition from iron-replete to iron-starved conditions [18]. In *S. cerevisiae*, studies have shown that Fra1 and Fra2 could form a heteroprotein complex with Grx3 and Grx4. Once assembled, the resulting heteromeric complex is involved in the signaling of excess iron to the regulator Aft1, triggering its inactivation [34], [35]. Although in the case of Aft1, the Fra1/2-mediated complex is needed for its iron-dependent inhibition (instead of an iron starvation-dependent inhibition), we assessed whether Fra1- and BolA-like homologs in *S. pombe* would be required to inactivate Fep1 function under low iron conditions. On the basis of amino acid sequence similarity, putative Fra1 (*SPAC22G7.01c*) and BolA-like homologs (Fra2 [*SPAC8C9.11*], Fra3 [*SPCC4B3.11c*], and Uvi31 [*SPBC16E9.06c*]) were identified from the *S. pombe* Genome Project. Single (*fra1Δ*, *fra2Δ* and *fra3Δ*), double (*fra1Δ fra2Δ*, *fra1Δ fra3Δ*, *fra2Δ fra3Δ*) and triple (*fra1Δ fra2Δ fra3Δ*) mutant strains were created and then used to analyze *fio1^+^* gene expression as a function of iron availability. In wild-type strain (used as a control), steady-state levels of *fio1^+^* were repressed under iron-replete conditions and induced under basal and low-iron conditions (Fig. 1A). In contrast, mutant cells harboring a deletion of *fra2^+^* (*fra2Δ*, *fra1Δ fra2Δ*, *fra2Δ fra3Δ*, and *fra1Δ fra2Δ fra3Δ*) exhibited low levels of *fio1^+^* transcripts, even in the presence of the iron chelator Dip (Fig. 1A). *fra1Δ* and *fra3Δ* single mutants as well as *fra1Δ fra3Δ* double mutant cells behaved similarly when compared to wild-type strain, exhibiting proper iron-dependent regulation of *fio1^+^* gene expression (Fig. 1A). We also tested a second series of mutants in which a third BolA-like gene, *uvi31^+^* was disrupted in combination with a deletion of either or both *fra2^+^* and *fra3^+^* (Fig. 1B). Using the aforementioned mutant strains, we found that only those cells carrying a *fra2^+^*-disrupted allele (*fra2Δ*, *fra2Δ uvi31Δ*, *fra2Δ fra3Δ uvi31Δ*) exhibited a lack of transcriptional induction of *fio1^+^* in response to iron starvation. The regulation of *fio1^+^* in the *uvi31Δ* and *fra3Δ uvi31Δ* mutant strains was comparable to that observed in wild-type strain (Fig. 1B). Given the fact that the elimination of Fra2 led to a constitutive repression of *fio1^+^* expression, we concluded that Fra2 plays an important role in the inactivation of Fep1 under iron-limiting conditions. Based on this observation, we analyzed steady-state mRNA levels of *fra2^+^* to determine whether they were regulated as a function of iron availability. Results showed that *fra2^+^* transcript levels were constitutive and unchanged by cellular iron status (Fig. 1C). Because the absence of Fra2 led to a constitutive repression of a gene (*fio1^+^*) involved in high-affinity iron uptake, *fra2Δ* mutant cells were spotted onto a medium depleted of iron by addition of the iron chelator Dip. Results consistently showed that *fra2Δ* cells exhibited poor growth on low iron medium in comparison to wild-type cells (Fig. 1D). Conversely, cells expressing a *fra2^+^-Myc_13_* fusion gene integrated at the chromosomal locus of *fra2^+^* regained the ability to grow in medium containing Dip (Fig. 1D). A *php4Δ* mutant was used as a control strain as it was previously shown to be unable to grow on low iron medium [7]. Based on these results, we concluded that although the expression of *fra2^+^* was invariable, its presence was required for proper iron starvation-dependent induction of *fio1^+^* when cells were starved for iron.

### Fra2 participates in the inhibition of Fep1 function through a Php4-independent mechanism

**Figure 2:**
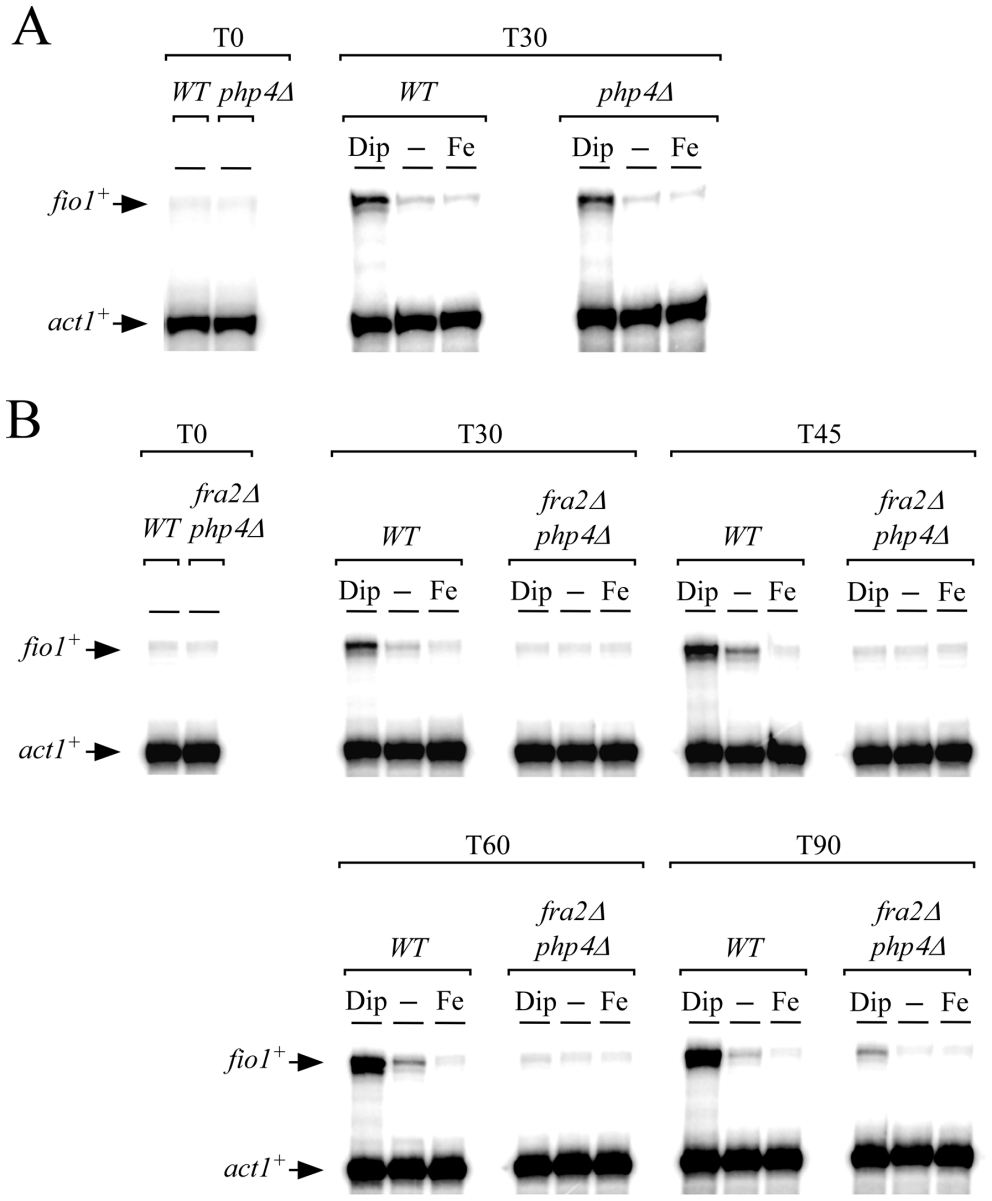
*fio1^+^* transcript levels are constitutively repressed in a *php4*Δ strain lacking the *fra2^+^* gene. *A*, *fio1^+^* steady-state mRNA levels were assessed in wild-type (*WT*) and *php4*Δ strains. Total RNA was extracted from culture aliquots and were incubated in the absence (-) or presence of Dip (250 µM) or FeCl_3_ (100 µM) for 0 and 30 min. For simplicity, the time point 30 min is shown since the mRNA signals detected (*fio1^+^* and *act1^+^*) at other time points (*e.g*. 45, 60 and 90 min) were sensibly identical. *B*, Isogenic wild-type (*WT*) and *fra2*Δ *php4*Δ strains were grown and samples of cultures were taken after 0, 30, 45, 60 and 90 min of Dip (250 µM) and FeCl_3_ (100 µM) treatment. As controls, cultures were left untreated (-) during the time course of the experiments. Total RNA was extracted and analyzed by RNase protection assays. Arrows indicate signals corresponding to *fio1^+^* and *act1^+^* mRNA steady-state levels. Results are representative of three independent experiments.

We have previously shown that *fep1^+^* mRNA levels were repressed under iron-limiting conditions [8]. This transcriptional down-regulation of *fep1^+^* expression is under the control of the negative regulatory subunit Php4 of the CCAAT-binding factor [8]. To ensure that the disruption of *fra2^+^* (*fra2Δ*) had no influence at the transcriptional level on the expression of *fep1^+^*, we created a *php4Δ fra2Δ* double mutant strain to unlink the iron starvation-dependent behavior of Fep1 from its transcriptional regulation by Php4. In the absence of Php4, *fep1^+^* transcript levels were constitutive and unaffected by changes in iron levels [8], the gene product (Fep1) could still be inactivated since *fio1^+^* transcripts were clearly induced in the presence of Dip (Fig. 2A).

Wild-type and *php4Δ fra2Δ* cells were precultured in the presence of FeCl_3_ (100 µM) to foster Fep1 activity and iron-dependent repression of *fio1^+^* expression. Cells were harvested at logarithmic phase, washed and resuspended in the same medium containing either Dip (250 µM), FeCl_3_ (100 µM) or were left without treatment for the indicated periods of time (Fig. 2B). Results showed that in wild-type cells treated with Dip, *fio1^+^* mRNA levels were up-regulated in response to iron starvation. The response occurred within 30 min and persisted for at least 90 min of Dip treatment (Fig. 2B). In contrast, *php4Δ fra2Δ* cells displayed very low *fio1^+^* transcript levels under all three conditions (Fig. 2B). Although a slight increase in *fio1^+^* transcription was observed after 90 min of Dip treatment in *php4Δ fra2Δ* cells, *fio1^+^* mRNA levels in the mutant strain were still 10.2+/−0.6-fold lower compared to the levels of *fio1^+^* observed in wild-type strain under the same conditions. Analysis of a *php4Δ* strain lacking *fra2^+^* (*fra2Δ*) strengthened the interpretation that Fra2 acted as a negative regulator of Fep1 in iron-starved cells.

### *fio1^+^* mRNA levels are constitutively down-regulated in a Fep1-dependent manner in a *php4Δ* strain lacking *fra2^+^*

**Figure 3:**
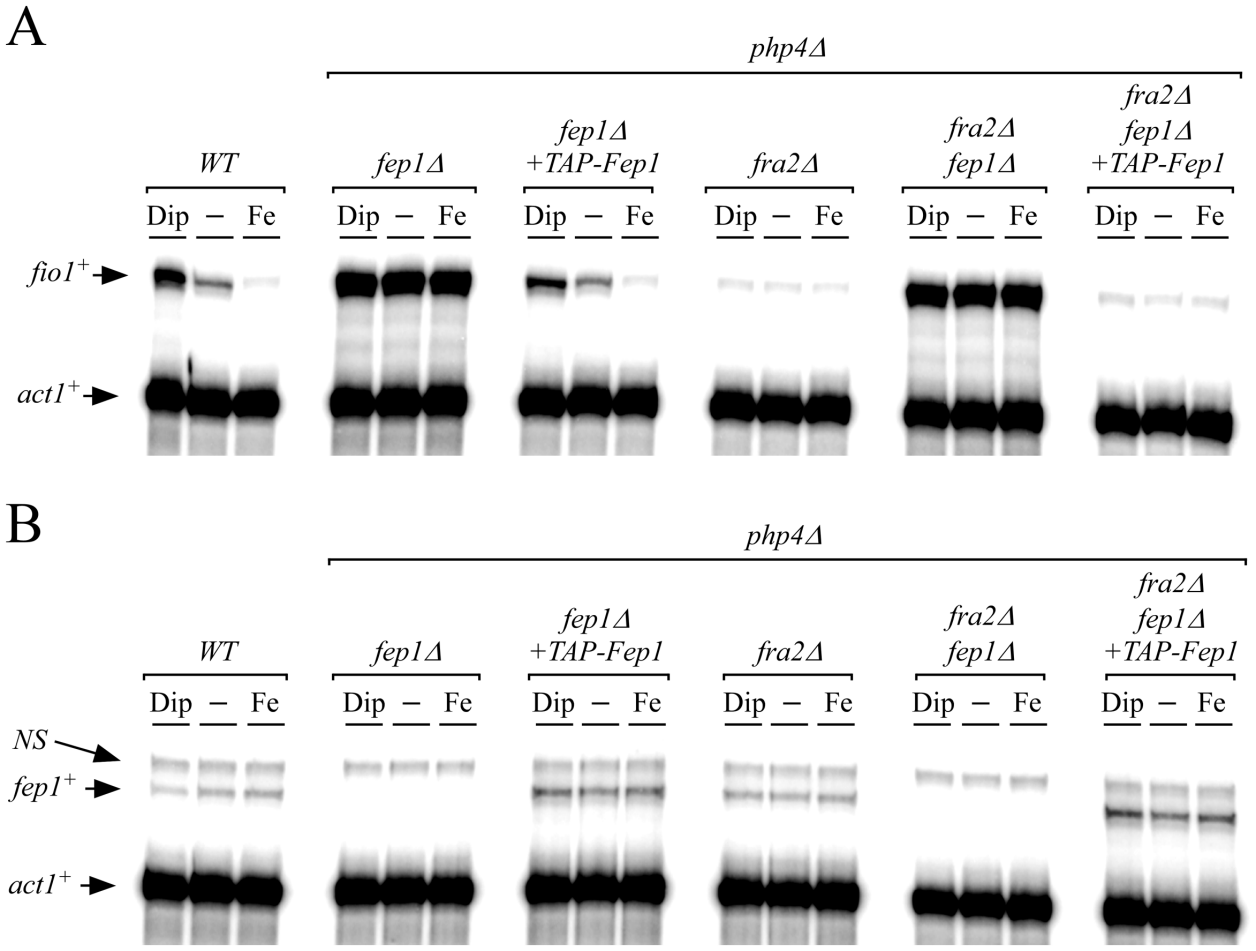
*fio1^+^* transcription is constantly repressed in a Fep1-dependent manner in *php4*Δ *fra2*Δ cells. *A*, RNase protection analysis of the *fio1^+^* and *act1^+^* transcript levels in wild-type (*WT*), *php4*Δ *fep1*Δ, *php4*Δ *fra2*Δ, and *php4*Δ * fep1*Δ *fra2*Δ strains exposed to 250 µM Dip or 100 µM FeCl_3_ or left untreated (-). When indicated, *php4*Δ *fep1*Δ and *php4*Δ *fep1*Δ *fra2*Δ cells were transformed with an integrative plasmid that encoded a functional *TAP-tagged fep1^+^* allele. *B*, Total RNA from cultures incubated under conditions described in *panel A* were used to probe steady-state levels of *fep1^+^* and *act1^+^* mRNAs. NS, non-specific signal. The results are representative of three independent experiments.

To obtain evidence of the requirement of Fep1 in repressing *fio1^+^* transcription in the absence of Fra2 (*fra2Δ*), we created a *php4Δ fra2Δ fep1Δ* triple mutant strain. In this case, *fio1^+^* transcript levels were strongly and constitutively expressed (14.4+/−0.8-fold as compared to basal levels of *fio1^+^* mRNA observed in wild-type strain) and were not regulated by cellular iron status (Fig. 3A). In contrast, when a functional *TAP-fep1^+^* fusion allele was returned by integration in *php4Δ fra2Δ fep1Δ* cells, *fio1^+^* transcription was strongly repressed under basal, iron-replete and iron-depleted conditions. The reduced levels of *fio1^+^* were similar to that of the *php4Δ fra2Δ* double mutant cells, confirming the predominant role of Fep1 in repression of *fio1^+^* expression (Fig. 3A). In a wild-type strain or in a *php4Δ fep1Δ* mutant strain in which a functional *TAP-fep1^+^* gene was reintroduced, *fio1^+^* mRNA levels were reduced 3.4+/−0.6-fold under high iron conditions compared to levels under basal conditions (Fig. 3A). In contrast, *fio1^+^* transcript levels were induced 5.1+/−0.9-fold in the presence of Dip since there was an endogenous *fra2^+^* gene in these strains (Fig. 3A). As previously shown, *fio1^+^* mRNA levels in both untreated and iron-treated *php4Δ fep1Δ* cells were highly derepressed and were even higher to the levels observed in iron-starved wild-type cells (Fig. 3A).

In parallel, aliquots of RNA samples used to determine *fio1^+^* steady-state mRNA levels were analyzed to assess steady-state levels of *fep1^+^* mRNA. Results showed that *fep1^+^* transcript levels were slightly decreased (1.7+/−0.3-fold) in iron-starved wild-type cells in comparison to their levels in untreated cells (Fig. 3B). The reduction in *fep1^+^* mRNA levels was dependent on Php4 since it was not observed in *php4Δ* strains in which endogenous *fep1^+^* [8] or a functional *TAP-fep1^+^* gene was expressed (Fig. 3B). Collectively, these results showed that Fep1 failed to respond to low iron levels in the absence of Fra2 that led to constitutive activation of Fep1 and constant repression of *fio1^+^*.

### Deletion of *fra2^+^* results in a sustained association of Fep1 with the *fio1^+^* promoter *in vivo*

**Figure 4:**
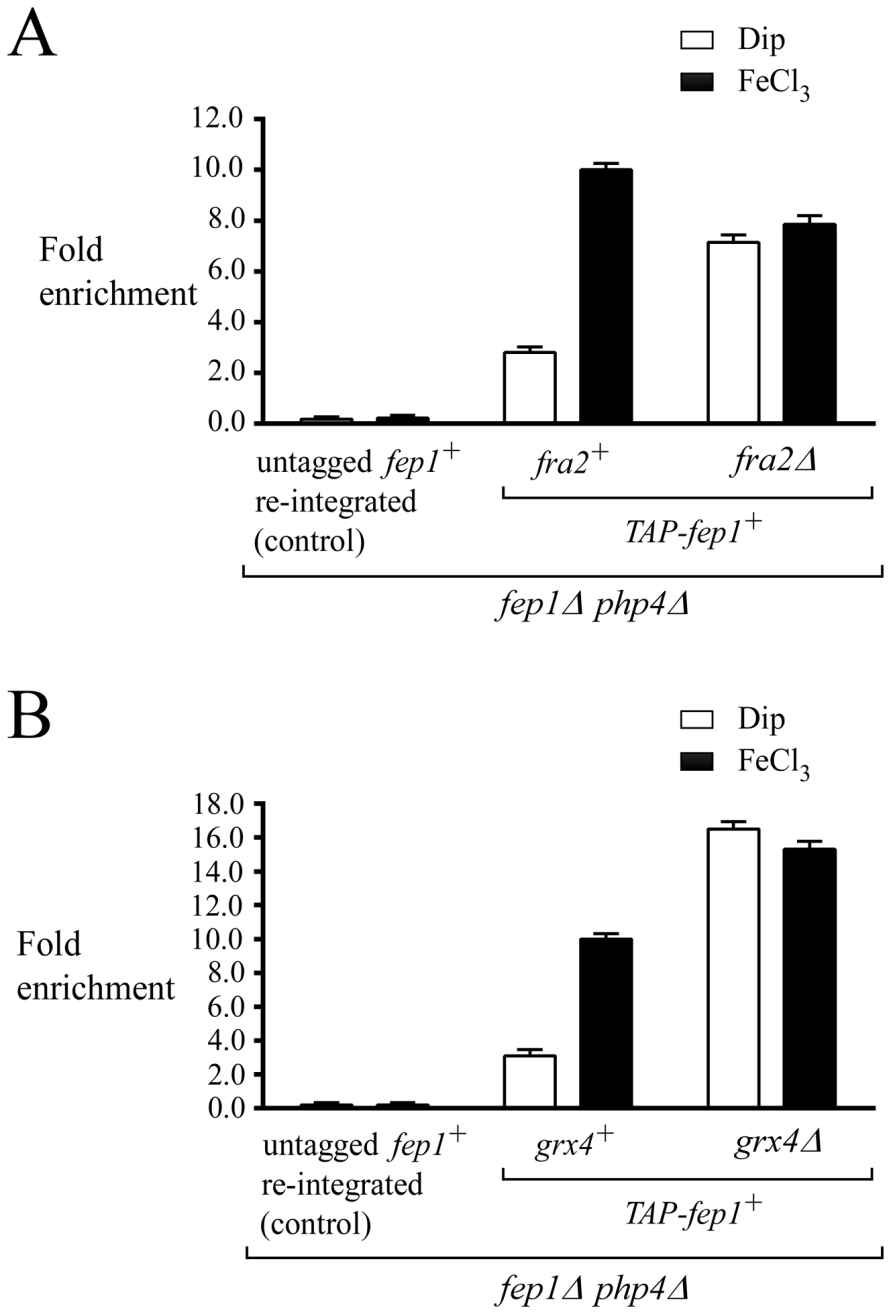
Iron starvation-dependent dissociation of Fep1 from its target gene promoter *fio1^+^* requires Fra2. *A*, ChIP analysis of the *fio1^+^* promoter in a *fep1*Δ * php4*Δ double mutant or a *fep1*Δ * php4*Δ * fra2*Δ triple mutant strain harboring an integrated untagged or TAP-tagged *fep1^+^* allele. Cells were grown to logarithmic phase in the presence of FeCl_3_ (100 µM), and then incubated in the presence of 250 µM Dip or 250 µM FeCl_3_ (Fe) for 30 min. Chromatin was immunoprecipitated using an anti-mouse IgG antibodies and a specific region of the *fio1^+^* promoter was analyzed by qPCR to determine Fep1 occupancy. Binding of TAP-Fep1 to the *fio1^+^* promoter was calculated as the enrichment of a specific *fio1^+^* promoter region relative to a 18S ribosomal DNA coding region in which no GATA box was present. ChIP data were calculated as values of the largest amount of chromatin measured (fold enrichment). Results are shown as the average +/− standard deviation of a minimum of three independent experiments. *B*, ChIP analysis was performed on the *fio1^+^* promoter in *fep1*Δ *php4*Δ or *fep1*Δ * php4*Δ * grx4*Δ cells expressing an untagged or TAP-tagged *fep1^+^* allele. This analysis was performed as a control experiment because it is known that deletion of the *grx4^+^* gene leads to constitutive promoter occupancy by Fep1. ChIP data were calculated and presented as described in *panel A*.

To test whether Fep1 constitutively occupied the *fio1^+^* promoter in the absence of Fra2, a ChIP approach was used to assess the levels of *fio1^+^* promoter occupancy by a functional TAP-Fep1 when *fra2^+^* was deleted. To disengage Fra2-dependent effect on behavior of TAP-Fep1 from its transcriptional regulation by Php4, *php4Δ fep1Δ* double and *php4Δ fep1Δ fra2Δ* triple mutant strains were used to ensure a constitutive transcriptional expression of *TAP-fep1^+^*. Cells were precultured in the presence of FeCl_3_ (100 µM) to ensure that TAP-Fep1 occupied the *fio1^+^* promoter. Subsequently, logarithmic-phase cells were harvested, washed and resuspended in the same medium containing either Dip (250 µM) or FeCl_3_ (250 µM), for 30 min. Results showed that TAP-Fep1 occupied the *fio1^+^* promoter at high levels in iron-replete *php4Δ fep1Δ* and *php4Δ fep1Δ fra2Δ* cells with 10.0+/−0.3- and 7.9+/−0.4-fold enrichments, respectively, relative to a 18S ribosomal DNA coding region in which no GATA box is present (used as a negative control) (Fig. 4A) [41]. Importantly, in the case of iron-starved *php4Δ fep1Δ fra2Δ* cells, the association of TAP-Fep1 with the *fio1^+^* promoter was elevated, with an occupancy 2.6+/−0.3-fold higher than that observed in *php4Δ fep1Δ fra2^+^* cells grown under the same conditions (Fig. 4A). This result was reminiscent of that observed in *php4Δ fep1Δ grx4Δ* cells grown under low levels of iron (Fig. 4B) [18]. When *grx4^+^* was deleted (*php4Δ fep1Δ grx4Δ*), the association between TAP-Fep1 and the *fio1^+^* promoter became sustained in a manner similar to that of a *fra2Δ* deletion (*php4Δ fep1Δ fra2Δ*), regardless of iron availability (Fig. 4B). In the absence of Grx4, the association of TAP-Fep1 with the *fio1^+^* promoter was highly enriched under both low (16.5+/−0.3-fold) and high (15.3+/−0.4-fold) iron conditions (relative to a 18S ribosomal DNA coding region in which no GATA box is present). These levels of enrichment were elevated in comparison with those of a *php4Δ fep1Δ* strain expressing an untagged *fep1^+^* allele in which case there was no enrichment. In the presence of Grx4, *php4Δ fep1Δ* cells expressing *TAP-fep1^+^* exhibited 3.2+/−0.3-fold higher levels of *fio1^+^* promoter DNA immunoprecipitated when chromatin was prepared from cells grown in the presence of iron than from cells cultured in the presence of Dip (Fig. 4B). Taken together, data showed that a deletion of *fra2^+^* (*fra2Δ*) phenocopies a *grx4Δ* deletion, and that resulted in a constitutive ability of Fep1 to bind to the *fio1^+^* promoter *in vivo*.

### Cellular localization of Fra2

**Figure 5:**
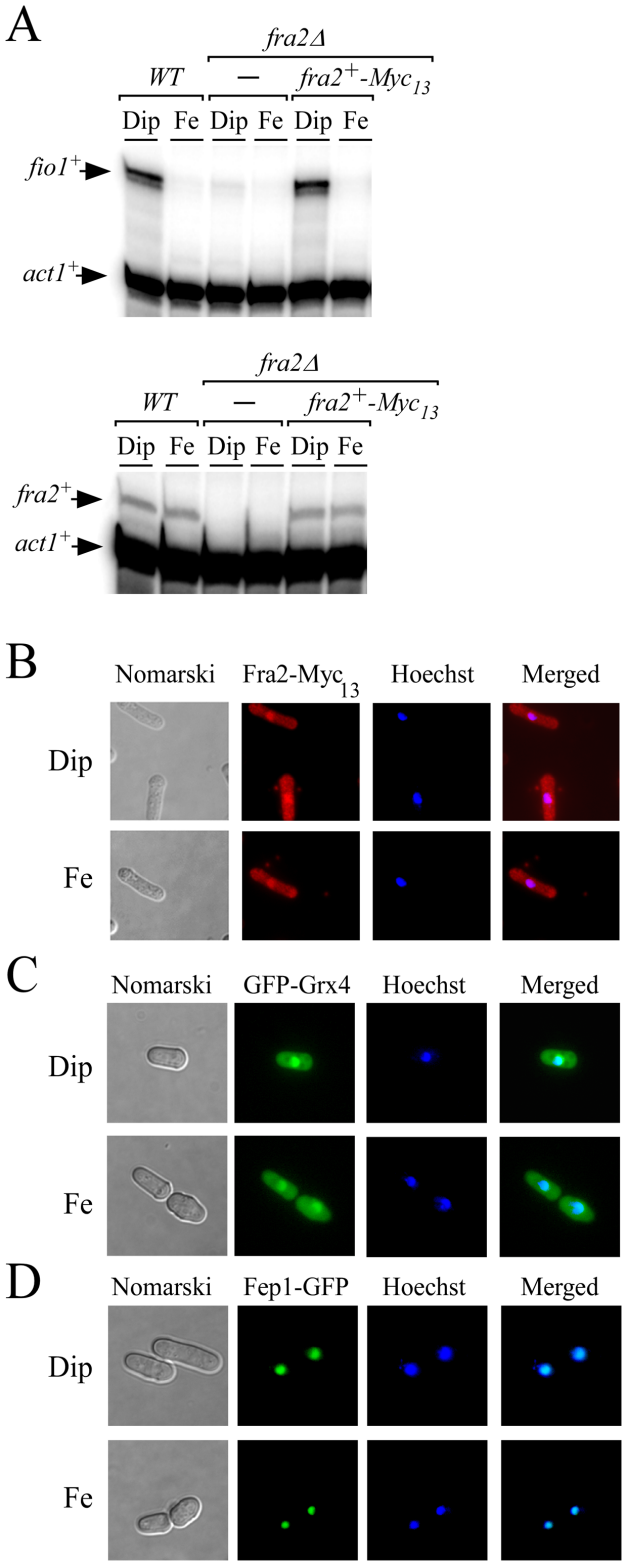
Fra2, Grx4, and Fep1 co-localize in the nucleus. *A*, *fio1^+^*, *fra2^+^* and *act1^+^* mRNA steady-state levels were determined in a wild-type (*WT*) and a *fra2*Δ disrupted strain in which either an empty vector alone (-) or a wild-type copy of the *fra2^+^-Myc_13_* allele was returned. Results are representative of three independent experiments. *B-D*, Cells expressing Myc_13_-tagged Fra2 (*panel B*), GFP-tagged Grx4 and Fep1 proteins (*panels C and D*) were treated with Dip (250 µM) or FeCl_3_ (Fe, 100 µM) for 30 min. Cells were analyzed by fluorescence microscopy for the presence of GFP (center left) and Hoechst stain (center right). Merged images are shown in the far right panels, whereas Nomarski pictures are depicted in the far left panels.

To further investigate how Fra2 participated in the inactivation of Fep1 function in response to iron starvation, we generated cells containing a *fra2^+^-Myc_13_* fusion gene integrated at the chromosomal locus of *fra2^+^*, resulting in the production of Fra2-Myc_13_ (Fig. 5). To make sure that the in-frame *Myc_13_* insertion did not interfere with Fra2 function, cells expressing the *fra2^+^-Myc_13_* allele were analyzed for their ability to activate *fio1^+^* transcript levels in response to low iron, which is an evidence of inhibition of Fep1 function. Expression of Fra2-Myc_13_ resulted in cells that exhibited iron starvation-dependent induction of *fio1^+^* expression in a manner similar to that of wild-type strain (Fig. 5A). In contrast, deletion of *fra2^+^* (*fra2Δ*) resulted in sustained repression of *fio1^+^* mRNA and lack of response to low iron conditions (Fig. 5A). When expressed, the *fra2^+^-Myc_13_* allele was present at low levels, in a manner comparable to that of wild-type strain (Fig. 5A). We next examined cellular localization of Fra2-Myc_13_ in cells that had been incubated in the presence of Dip or FeCl_3_. Under both conditions, Fra2-Myc_13_ fluorescence was detected in cytosolic and nuclear regions (Fig. 5B and data not shown). In most cells, a significant proportion of Fra2-Myc_13_ fluorescence co-localized with Hoechst staining, thus confirming that Fra2-Myc_13_ can localize to the nuclear compartment (Fig. 5B and data not shown). As previously reported [18], [21] and under the same culture conditions, *grx4Δ* cells expressing a functional *GFP*-tagged *grx4^+^* allele produced a fluorescent signal in nuclear and cytosolic regions, with a predominant signal associated with the nucleus (Fig. 5C). In the case of Fep1, functional Fep1-GFP was exclusively detected in nuclei, independently of the levels of iron (Fig. 5D) [9]. Indeed, Fep1-GFP fluorescence co-localized with DNA-staining Hoechst dye (Fig. 5D). These observations led to the conclusion that Fra2 localized throughout the cytoplasm and nuclei of cells, leaving open the possibility that the protein interacted with Grx4 in subcellular regions, and with Fep1 in the nucleus.

### Fra2, Grx4, and Fep1 associate as a heteroprotein complex

**Figure 6:**
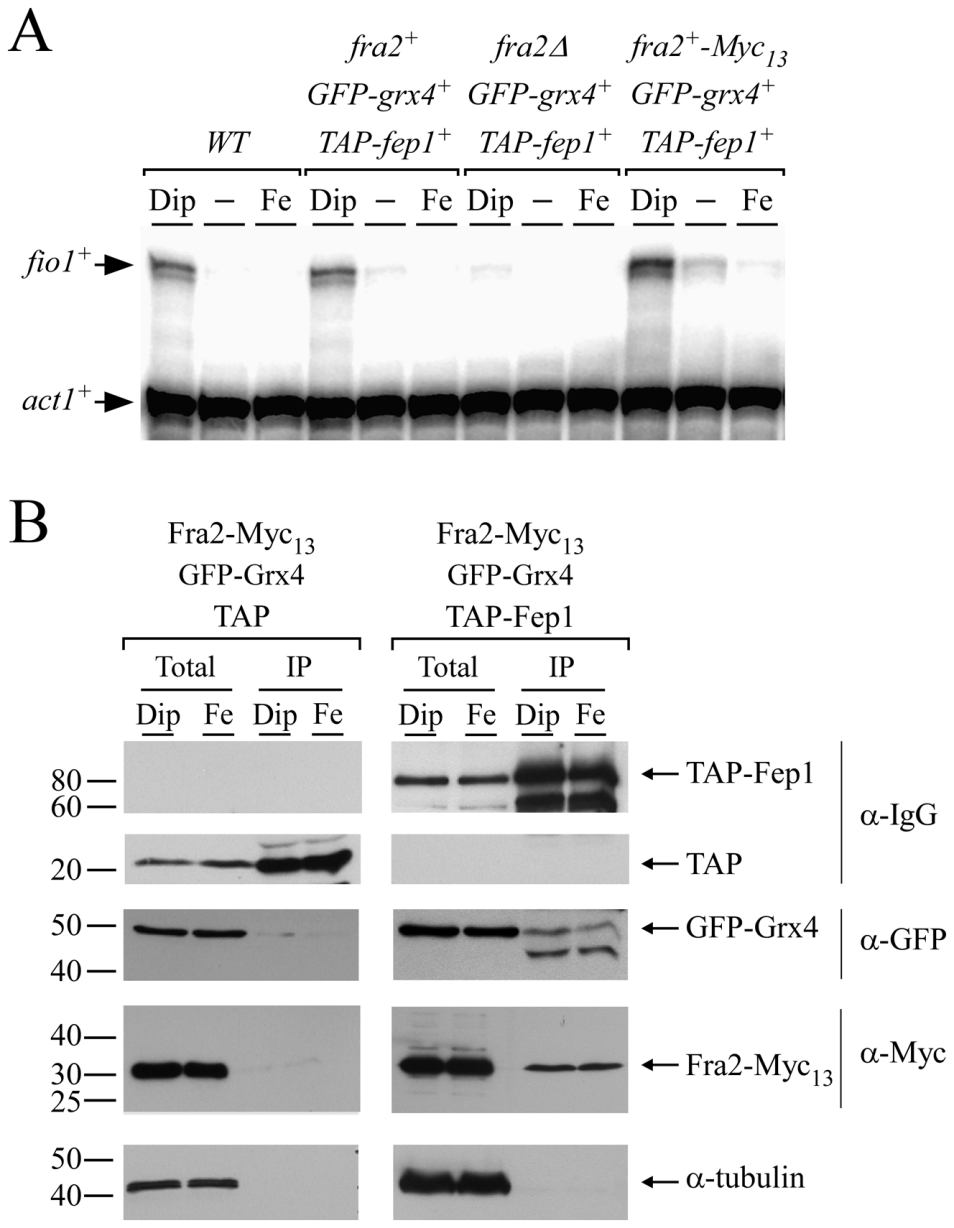
Fra2 and Grx4 interact with Fep1 in *S. pombe*. *A*, RNase protection analysis of *fio1^+^* and *act1^+^* transcript levels in *fep1Δ grx4Δ* cells harboring *TAP-fep1^+^* and *GFP-grx4^+^* alleles in the absence (*fra2Δ*) or presence of *fra2^+^* or *fra2^+^-Myc_13_* allele. The parent FY435 (*WT*) was used as a positive control for repression and induction of *fio1^+^* expression under iron-replete and iron starved conditions, respectively. (-), untreated cells. Results are representative of three independent experiments. *B*, Cells expressing TAP-tagged Fep1, GFP-tagged Grx4 and Myc_13_-tagged Fra2 or TAP alone, GFP-tagged Grx4 and Myc_13_-tagged Fra2 were incubated in the presence of Dip (250 µM) or FeCl_3_ (Fe; 100 µM). Extracts (Total) were subjected to immunoprecipitation (IP) using IgG-Sepharose beads. The bound proteins were eluted and analyzed by immunoblot assays using a mouse anti-GFP antibody (α-GFP) and an anti-Myc antibody (α-Myc). A portion of the total cell extracts (∼2%) was included to ascertain the presence of proteins prior to chromatography. As additional controls, aliquots of whole-cell extracts and bound fractions were probed with anti-mouse IgG antibody (α-IgG) and anti-tubulin antibody (α-tubulin). The positions of the molecular weight of protein standards (in kDa) are indicated on the left-hand side.

Given the fact that Fra2 was required for inactivation of Fep1 in response to iron deprivation, protein-protein interaction assays were carried out to investigate the possibility that Fra2 physically associated with Fep1 in *S. pombe*. Cells co-expressing *fra2^+^-Myc_13_*, *GFP-grx4^+^*, and *TAP-fep1^+^* alleles conferred iron-dependent regulation of *fio1^+^* expression in a manner similar to that of wild-type Fra2, Grx4 and Fep1 in the parental strain (Fig. 6A). Results showed that *fio1^+^* transcript levels were upregulated in the presence of Dip compared to levels of transcripts detected under basal and iron-replete conditions (Fig. 6A). Thus, the three fusion proteins (Fra2-Myc_13_, GFP-Grx4 and TAP-Fep1) were functionally competent. TAP pull-down experiments were performed in cells co-expressing *TAP-fep1^+^*, *GFP-grx4^+^* and *fra2^+^-Myc_13_* or *TAP*, *GFP-grx4^+^* and *fra2^+^-Myc_13_* alleles in the presence of the iron chelator Dip or iron (Fig. 6B). Total cell extracts were incubated in the presence of IgG-Sepharose beads that selectively bound (unfused) TAP or TAP-tagged Fep1. In the latter case, it allowed an enrichment of Fep1 and detection of potential interacting partners. Western blot analysis of the proteins retained by the beads (bound fraction) using anti-Myc and anti-GFP antibodies revealed that both Fra2-Myc_13_ and GFP-Grx4 were present in the immunoprecipitate fraction of cells grown under iron-limiting and iron-replete conditions (Fig. 6B). In contrast, and regardless of the iron levels, neither Fra2-Myc_13_ nor GFP-Grx4 was significantly found in the bound fraction of cells expressing TAP alone (Fig. 6B). Fractionation of the pull-down experiments was validated using an antibody directed against α-tubulin. Results showed that α-tubulin was present in total cell extracts but not in the retained protein fraction (Fig. 6B). To assess the steady-state protein levels of TAP-Fep1, Western blot analyses of both the protein preparations and the bound fractions were carried out using anti-IgG antibody (Fig. 6B). Taken together, TAP pull-down experiments showed that TAP-Fep1 and both Fra2-Myc_13_ and GFP-Grx4 interacted with each other to form a stable heteroprotein complex that was present in whole-cell extracts, irrespective of iron status.

### Fra2 associates with Fep1 in the nucleus

**Figure 7:**
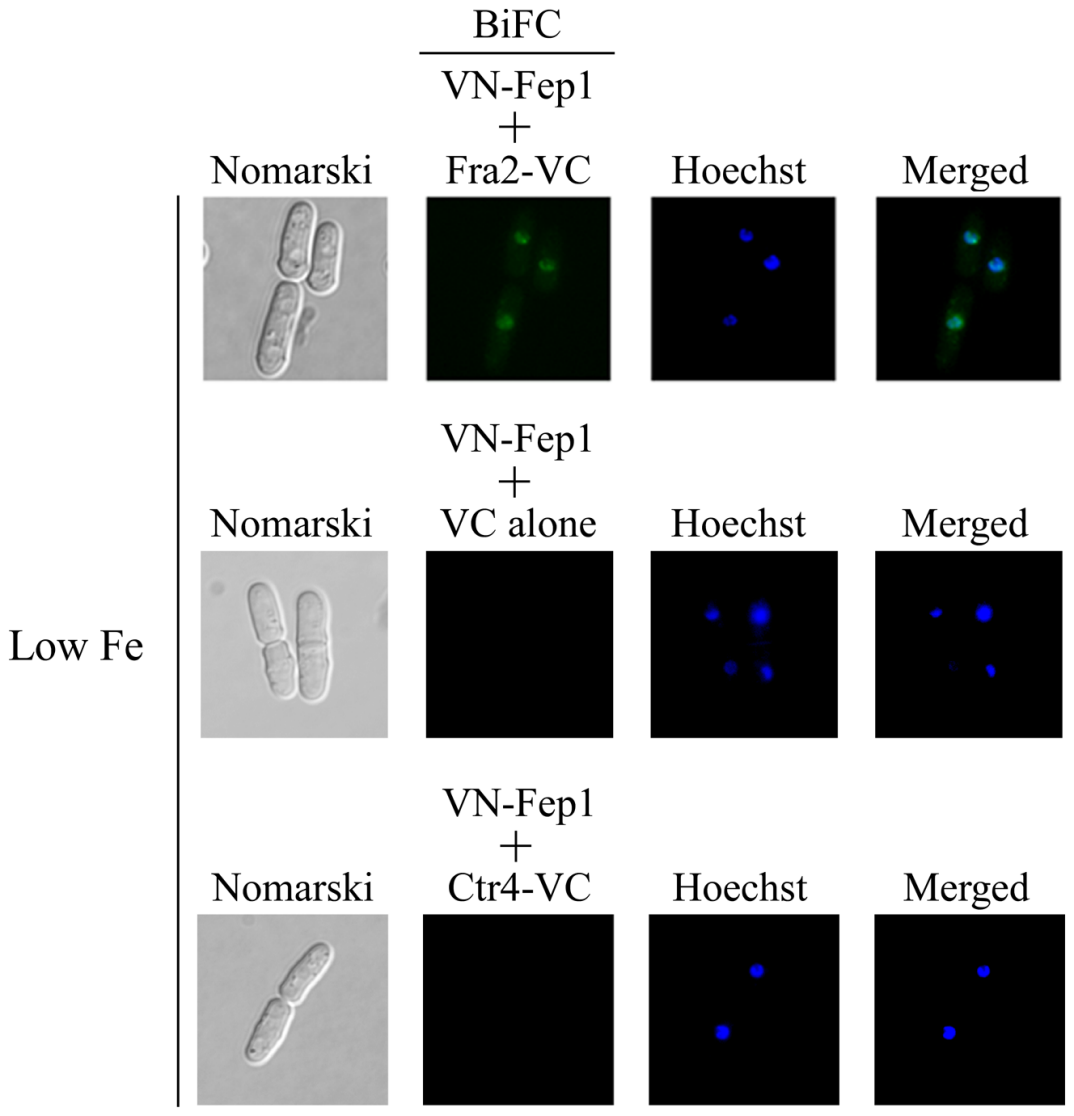
BiFC signal of VN-Fep1 and Fra2-VC fusion proteins in the nuclei. Cells co-expressing VN-Fep1 and Fra2-VC, VN-Fep1 and VC alone, or VN-Fep1 and Ctr4-VC were visualized by fluorescence microscopy using BiFC (center left) and Hoechst stain (center right). The merged images are shown in the far right panels. Nomarski optics were used to examine cell morphology (far left panels). For simplicity, images were taken from iron-starved cells because fluorescent images from iron-replete cells were identical.

Given that Fra2 associates with Fep1 in a heteroprotein complex in coimmunoprecipitation assays, we investigated their capacity to interact *in vivo* by using a BiFC approach in fission yeast. In these experiments, Venus N-terminal fragment (VN) and Venus C-terminal fragment (VC) were fused to the N- and C-terminal portions of Fep1 and Fra2, respectively. Under both iron starvation and iron-replete conditions, the VN-tagged Fep1 and VC-tagged Fra2 produced BiFC signals, indicating that Fep1 and Fra2 were forming heteromeric complexes (Fig. 7 and data not shown). Importantly, VN-Fep1-Fra2-VC fluorescent complexes were seen primarily in nuclei (Fig. 7 and data not shown). Fluorescence was observed in cells co-expressing VN-Fep1 and Fra2-VC fusion proteins but not in cells expressing only one of the fusion proteins (Fig. 7 and data not shown). Furthermore, there was an absence of BiFC signal in cells co-expressing two unrelated proteins harboring the N- and C-terminal fragments of Venus, such as VN-Fep1 and Ctr4-VC [47] (Fig. 7). Taken together, these results indicated that the interaction between VN-Fep1 and Fra2-VC fusion proteins occurred in the nuclei of living cells.

## Discussion

When *S. pombe* cells undergo transition from low to high iron, Fep1 represses the expression of several genes involved in the acquisition of iron. In contrast, conditions of low concentrations of iron favor Fep1 inactivation and dissociation from its target gene promoters [9]. The iron limitation-dependent inhibition of Fep1 occurs at multiple levels. First, Fep1 expression is down-regulated by the low-iron-responsive sensor Php4, which is a specialized subunit of the CCAAT-binding factor. Following its association with the Php2/Php3/Php5 heterotrimer, Php4 blocks *fep1^+^* gene transcription. A second mechanism of Fep1 inactivation takes place at the posttranscriptional level and requires the monothiol glutaredoxin Grx4. The previous finding that Grx4 is an inhibitory partner of Fep1 was rather surprising since monothiol glutaredoxins are known to inhibit iron-regulatory transcription factors in response to excess iron but not to iron starvation [4]. In *S. cerevisiae*, the iron-regulatory transcription factor Aft1 induces genes of the iron regulon in response to low iron, whereas its transcriptional activity is inhibited under iron-replete conditions. Iron-dependent inhibition of Aft1 function requires the presence of different molecular components, including proteins involved in mitochondrial iron-sulfur cluster biosynthesis, glutathione, Fra1 and Fra2 proteins, and monothiol glutaredoxins Grx3 and Grx4 [34]. When mitochondrial iron-sulfur cluster or glutathione biosynthesis is defective or when Grx3 and Grx4 or Fra2 are absent, Aft1 constitutively activates transcription of its target genes, irrespective of iron status. In *S. pombe*, cells defective in glutathione biosynthesis or deficient in Grx4 (*grx4Δ*) exhibit markedly decreased transcription of genes encoding iron-using proteins as a result of constitutively active Php4 [8], [28]. Although Aft1 is a transcription activator and, in contrast, Php4 is a repressor, both proteins are active when iron levels are low. Consequently, to be able to sense the iron inhibitory signal, Aft1 and Php4 may use common cellular components along their transduction pathways. To further explore the iron-signaling pathway, we examined the effect of deleting the *fra2^+^* gene on Php4 activity. Unexpectedly, cells lacking Fra2 (*fra2Δ*) gave rise to normal expression levels of iron-using genes under iron-replete conditions, revealing that Php4 repressive function was inhibited in the absence of Fra2 (data not shown). Expression profiles of Php4 target genes in *fra2Δ* cells were comparable to those of wild-type cells. Under both basal (untreated) and iron-replete conditions, transcript levels of Php4 target genes were readily detectable. They were more abundant compared to transcript levels observed under low iron conditions (data not shown). Similarly, additional mutants harboring single or multiple deletions of *fra1^+^*, *fra2^+^*, *fra3^+^* and *uvi31^+^* showed no apparent defect on transcript levels of Php4 target genes (data not shown). Based on these observations, we concluded that iron-dependent inactivation of Php4 did not involve all the same molecular components as those of Aft1 in *S. cerevisiae*. Although Grx4 was required to inactivate Php4 and Aft1 in response to high levels of iron, *S. pombe* Fra1- and BolA-like proteins, such as Fra2, Fra3 and Uvi31, were not involved in the iron-signaling of the Php4-Grx4 complex. We have previously shown that deletion of *grx4^+^* (*grx4Δ*) resulted in Php4 being constitutively located in nuclei and being non-responsive to iron [28]. In the absence of Fra2 (*fra2Δ*), Php4 exhibited normal nucleocytoplasmic shuttling, being imported in the nucleus under low iron conditions and exported from the nucleus to the cytoplasm in response to iron (data not shown). This observation represented supporting evidence that Fra2 was not involved in the Grx4-sensing pathway that inactivates Php4 in response to iron.

These observations as well as the fact that *S. pombe* Grx4 serves as a regulator for two distinct iron-regulated transcription factors, Php4 and Fep1, under conditions of high and low levels of iron, led us to investigate whether Fra1- and BolA-like proteins were involved in the Grx4-sensing pathway that inactivates Fep1 in response to iron deficiency. Unexpectedly, results showed that Fra2 was required for inhibition of Fep1 function under iron starvation conditions. The elimination of Fra2 led to constitutive activation of Fep1 and binding to its target gene promoters *in vivo* (Fig. 4). In *fra2Δ* cells, Fep1 target genes were therefore invariably repressed and failed to respond to iron starvation. This result was reminiscent of that observed in *grx4Δ* cells cultivated under iron-limiting conditions [18]. Interestingly, we have observed that a double knockout of *grx4Δ* and *fra2Δ* gave constitutive low *fio1^+^* mRNA levels and constitutively high promoter occupancy by Fep1 (data not shown). Grx4 and Fep1 have been reported to mutually interact [18]. Furthermore, on the basis of studies in *S. cerevisiae*, it is known that Grx4 is also an interacting partner of Fra2 [35], [36]. We therefore hypothesized that Fra2 influenced Fep1 activity through its interaction with the Fep1-Grx4 complex. A combination of coimmunoprecipitation and BiFC assays revealed that Fra2 associated with Fep1 in an iron-independent manner. *In vivo* experiments using BiFC further showed that Fra2-Fep1 association was present in the nucleus. Although the mechanism by which Fra2 participates in the inactivation of Fep1 remains unclear, the physiological importance of this association deserves further investigation. In *S. cerevisiae*, His^103^ residue in Fra2 is an iron-sulfur cluster ligand in the Fra2-Grx3 complex that is required for *in vivo* iron-dependent inhibition of Aft1 activity [35]. Furthermore, Cys^66^ residue in Fra2 is necessary for Fra2-Grx3 association [35]. These two amino acid residues (Cys^66^ and His^103^) are highly conserved in *S. pombe* Fra2 and correspond to Cys^29^ and His^66^ residues, respectively. In connection with data reported here that Fra2 acted as an inhibitory partner for Fep1 under conditions of low iron levels, it would be informative to mutate His^66^ to test whether this mutation abolishes the ability of Fra2 to participate in the inactivation of Fep1 under low-iron conditions. If this proved not to be the case, it would suggest that Fra2 uses a different amino acid residue or region to negatively co-regulate Fep1 activity in response to iron deficiency. Similarly, substitution of Cys^29^ residue would yield information whether this amino acid residue is essential for interaction of Fra2 with the Grx4/Fep1 complex. In *S. cerevisiae*, Fra2 forms an iron-independent complex with Grx3/4 [34]. In the present study, Fra2 was also detected as an interacting partner of Fep1 under low and high iron conditions. The reason why Fra2 was required for inhibition of Fep1 is unknown at present. One possibility would be that Fra2 facilitates association between the GRX domain of Grx4 and the N-terminal portion of Fep1, leading to the inactivation of the Fep1 DNA binding domain. Under conditions of iron excess, Fra2 may also participate in the dissociation of the GRX domain from the N-terminal portion of Fep1 by forming a [2Fe-2S] Fra2-GRX domain heterodimer, allowing the N terminus of Fep1 to be free and available for binding to chromatin and repressing transcription of the target genes. Taken together, our findings revealed that Fra2 acts as a co-inhibitory partner for Fep1 when cells grow under are iron-limited conditions. These results highlight a novel function for Fra2, which is canonically known to play an inhibitory effect on low-iron-sensing factors when cells are iron-replete.
